# Mapping Ethical Artificial Intelligence Policy Landscape: A Mixed Method Analysis

**DOI:** 10.1007/s11948-024-00472-6

**Published:** 2024-03-07

**Authors:** Tahereh Saheb, Tayebeh Saheb

**Affiliations:** 1 School of Business, Menlo College, Atherton, CA USA; 2 Faculty of Law, Tarbiat Modares University, Tehran, Iran

**Keywords:** Artificial intelligence, Ethic, Government policies, Topic modeling, Content analysis

## Abstract

As more national governments adopt policies addressing the ethical implications of artificial intelligence, a comparative analysis of policy documents on these topics can provide valuable insights into emerging concerns and areas of shared importance. This study critically examines 57 policy documents pertaining to ethical AI originating from 24 distinct countries, employing a combination of computational text mining methods and qualitative content analysis. The primary objective is to methodically identify common themes throughout these policy documents and perform a comparative analysis of the ways in which various governments give priority to crucial matters. A total of nineteen topics were initially retrieved. Through an iterative coding process, six overarching themes were identified: principles, the protection of personal data, governmental roles and responsibilities, procedural guidelines, governance and monitoring mechanisms, and epistemological considerations. Furthermore, the research revealed 31 ethical dilemmas pertaining to AI that had been overlooked previously but are now emerging. These dilemmas have been referred to in different extents throughout the policy documents. This research makes a scholarly contribution to the expanding field of technology policy formulations at the national level by analyzing similarities and differences among countries. Furthermore, this analysis has practical ramifications for policymakers who are attempting to comprehend prevailing trends and potentially neglected domains that demand focus in the ever-evolving field of artificial intelligence.

## Introduction

The pursuit of creating a General AI system with cognitive abilities comparable to humans remains a significant challenge. While current AI methodologies have resulted in the development of specialized applications that can outperform humans in narrowly defined tasks, inherent limitations in these methods present barriers to the development of broadly intelligent systems (Hole & Ahmad, 2021). In response to the advancements in computational systems that simulate human perception and reasoning, many national governments have issued strategic guidelines to establish standards for AI innovation (Van Berkel et al., 2020). However, the rapid progress in AI and its potential for superintelligence and automation also brings about societal risks (Bostrom, 2020). It is widely recognized that AI can contribute to social injustice and inequality (Rafanelli, 2022). Consequently, several nations have taken measures to address the ethical and societal concerns raised by AI, aiming to manage its emergence in society and mitigate its potential downsides (Vesnic-Alujevic et al., 2020). There is an increasing focus among academics and practitioners on finding solutions to address the unjust societal consequences of artificial intelligence (Stix, 2021). Researchers have specifically addressed the issue of racial or gender disparities in training data (Ntoutsi et al., 2020; Saheb et al., 2021, 2022). They have also investigated the exploitation of algorithms and data processing for political purposes, including the dissemination of false news and misinformation (Taboada & Torabi Asr, 2019). These efforts highlight the importance of addressing biases and ethical concerns associated with AI systems to ensure fairness, equity, and responsible use of technology.

In the field of AI ethics, there has been a growing focus on legal guidance related to the development and application of artificial intelligence. Previous studies have examined various principles proposed by different stakeholders, including commercial entities, public institutions, and academic researchers. However, this study specifically concentrates on guidelines issued by national governments, narrowing the scope of analysis (Jobin et al., 2019). Previous research has primarily emphasized regulations from North American and European authorities (Pesapane et al., 2018). While international public institutions have also published their visions for ethical AI development, there are gaps in the existing literature in terms of comprehensive analysis that combines diverse viewpoints. Previous studies have either qualitatively examined specific principles (Hagendorff, 2020) or quantitatively analyzed them (Fjeld et al., 2020). This study aims to bridge this gap by employing an integrated mixed-methods analysis. Through a combination of quantitative and qualitative content analysis, the researchers seek to provide a comprehensive understanding of the similarities and differences in priorities by analyzing 57 policy documents from 24 countries across different regions. The study utilizes topic modeling and co-word analysis to identify prevailing and emerging themes at a global level. By integrating quantitative and qualitative findings, the researchers aim to develop a more comprehensive conceptual model that surpasses the limitations of individual approaches. Furthermore, the examination of variations in policy coverage among different nations allows for the exploration of geopolitical differences. This integrated methodology facilitates the mapping of dominant discourses and overlooked issues, contributing to an enhanced scholarly understanding of technological governance. Additionally, the study aims to provide practitioners with strategic intelligence regarding the global landscape of ethical AI policy. The findings serve as reference points for ongoing multilateral collaboration and the establishment of standards.

This study aims to address the subsequent inquiries:

Which topics are most frequently addressed in governmental documents concerning ethical AI?

Which topics are most prevalent in governmental publications concerning ethical AI?

What is each government agency's focal point in relation to these common ethical AI concerns and topics?

## Methodology

We used a mixed methodological approach to examine national policies on ethical AI in our investigation. Using the OECD's AI Observatory database (https://oecd.ai/en/), we identified 57 such policies promoted by 24 different countries and two distinct regions at the time of writing this article. Australia, Belgium, Canada, China, Dubai, the European Union, Finland, France, Germany, India, Ireland, Japan, Korea, Lithuania, Malta, Mexico, the Netherlands, New Zealand, Norway, Quad, Spain, Switzerland, Thailand, Turkey, the United Kingdom, and the United States are among these countries and regions. As a result, we gathered and analyzed these documents for the purposes of our research. We performed a topic modeling analysis using Provalis Research's WordStat (Davi et al., 2005), a tool that allows for both quantitative and qualitative evaluations of textual data. WordStat’s capabilities extend beyond data visualization to include text mining and content analysis. WordStat investigates textual data using a variety of algorithms and techniques, including keyword retrieval and keyword-in-context analysis. Keyword retrieval is a basic form of text analysis in which WordStat searches for specific words or phrases within the text (Jones, 2019). This is a fundamental form of pattern recognition in which the user specifies the pattern to be identified. WordStat can identify and display all instances of a specific word or phrase within its context, allowing researchers to interpret the usage of a word within its situational context.

**Fig. 1:**
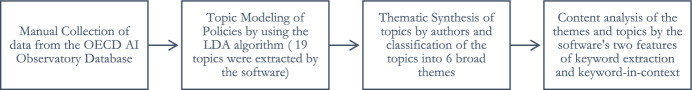
Research Methodology of the study

WordStat utilizes a statistical algorithm called Latent Dirichlet Allocation (LDA) to identify topics within a collection of documents (Jelodar, 2019). This approach enables the detection of recurring patterns and themes in a large corpus of text. In this particular study, the topic modeling analysis using LDA yielded nineteen distinct topics. After identifying these individual topics, the authors employed a thematic synthesis process based on the methodology outlined by Thomas (2008) to group these topics into larger clusters, as depicted in Fig. 1. This process involved several iterative steps. Initially, the authors familiarized themselves with the nineteen topics, gaining a comprehensive understanding of the ideas and concepts represented by each topic. The next step involved identifying preliminary themes that could encompass multiple topics. The authors examined the similarities, differences, and patterns among the topics to identify potential themes. In the third step, the authors reviewed and refined these preliminary themes by comparing them to the original topics and the entire dataset. This process included combining, splitting, discarding, or developing new themes as necessary. The fourth step focused on defining and naming the themes. Once the authors were satisfied with the number and composition of the themes, they provided clear definitions and appropriate names to each theme. Each theme formed a distinct and cohesive grouping that captured the essence of the topics encompassed within it. Finally, in the last step, the authors wrote the final analysis, connecting the topics together to form cohesive and insightful narrative.

Following the thematic synthesis, the authors conducted a qualitative analysis of policies using two techniques: keyword extraction and keyword-in-context features, both of which are essential tools for qualitative textual data analysis (Wiedemann, 2013). The process of extracting the most relevant words or phrases from a text is known as keyword extraction. This entailed identifying the most frequently used words and phrases, significant terms, and specific words and phrases of particular interest in the context of ethical AI policy data. WordStat employs complex algorithms that consider not only the frequency of a word, but also its distribution across different documents and co-occurrence with other words. This can help in identifying keywords that accurately reflect the content of the text. Keyword-in-context (KWIC) analysis examines words in the context in which they appear in the text. WordStat includes a KWIC feature that displays all occurrences of a specific word as well as the surrounding text. KWIC proved extremely helpful in understanding how a term is used in ethical AI policy analysis. To investigate how policy documents discuss "privacy," for example, we used the KWIC feature to identify all instances of the word "privacy" in the texts as well as the sentences in which it appears. This can provide valuable insights into the nuances of how a policy topic is addressed, insights that would otherwise be missed if we only looked at word frequencies. In policies, for example, are there specific rights, obligations, or exceptions associated with the term "privacy"? Is the term "privacy" used more frequently in specific contexts or circumstances? This feature helped the authors write the analysis section and suggest missing activities and topics.

## Results

### Identified Topics

The topic modeling analysis resulted in the identification of the following 19 topics: responsible AI systems, safety of autonomous vehicles, transparency, trust and accountability, human autonomy and social justice, common goods and democratic values, informed consent and GDPR, children online safety, machine assisted human decisions, automated decision making, algorithmic law enforcement, civil society participation, responsible private sector and targeted advertisement, high risk assessment and impact analysis, controllers of personal data processing, privacy by design, ethical AI talent workforce, data models and training data, explainable AI and bias, discrimination and fairness of algorithms.

**Fig. 2:**
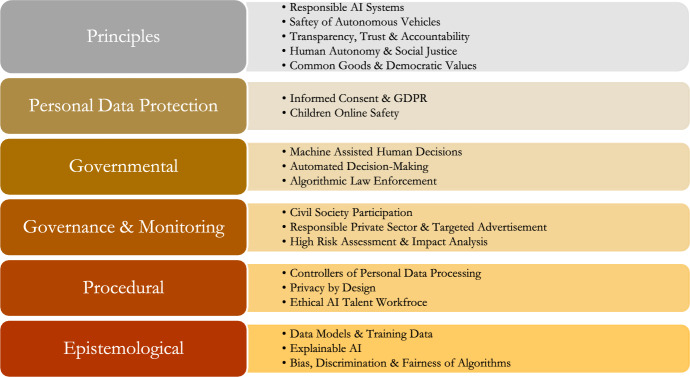
General themes and their corresponding topics. Themes were extracted by authors from qualitative analysis and topics were uncovered by topic modeling analysis

**Fig. 3:**
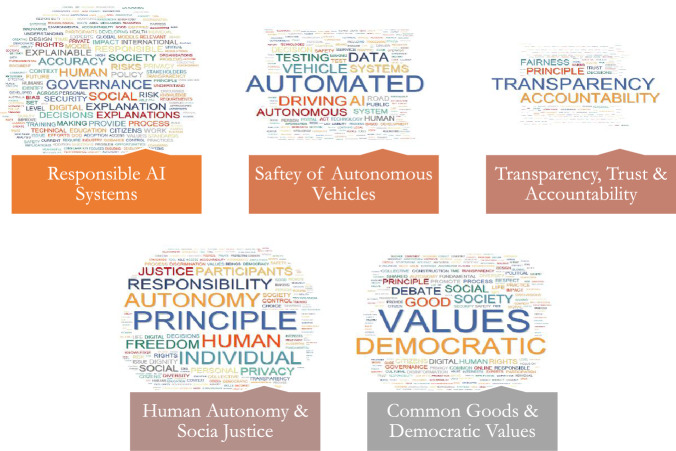
Word Cloud of Topics related to the PRINCIPLES Dimension

**Fig. 4:**
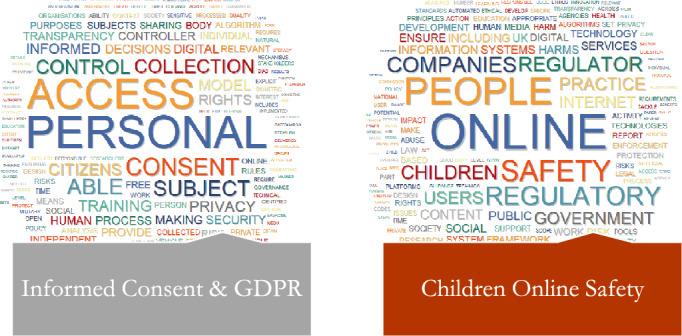
Word Cloud of Topics related to the PERSONAL DATA PROTECTION Dimension

**Fig. 5:**
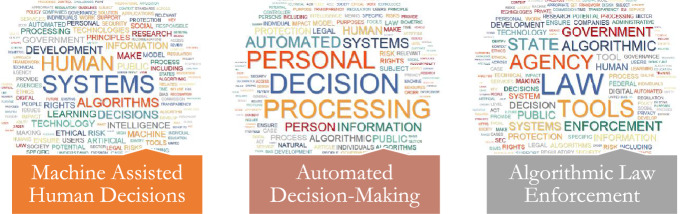
Word Cloud of Topics related to the GOVERNMENTAL Dimension

**Fig. 6:**
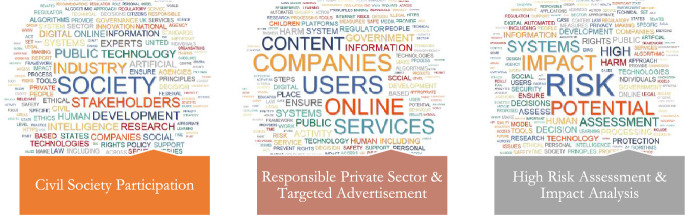
Word Cloud of Topics related to the GOVERNANCE & MONITORING Dimension

**Fig. 7:**
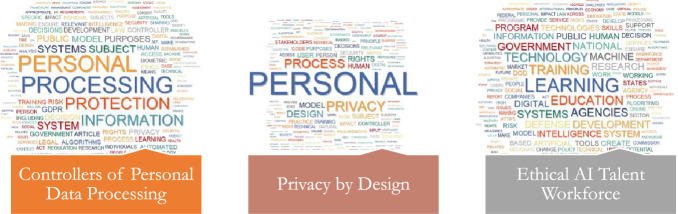
Word Cloud of Topics related to the PROCEDURAL Dimension

**Fig. 8:**
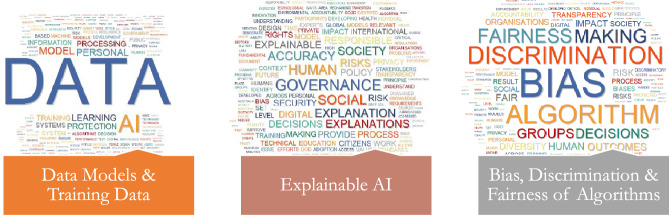
Word Cloud of Topics related to the EPISTEMOLOGICAL Dimension

**Fig. 9:**
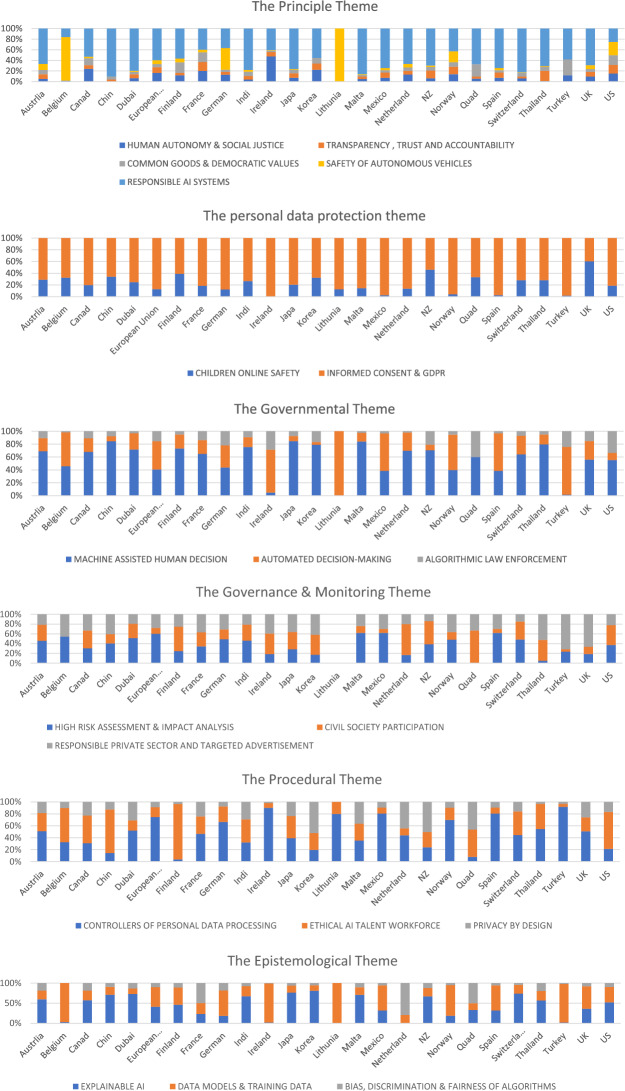
Global Distribution of the six ethical AI themes

Following the thematic synthesis procedure, the authors classified the 19 topics into 6 primary themes (Figs. 2, 3, 4, 5, 6, 7, 8, 9): Principles (five topics), Personal Data Protection (two topics), Governmental (three topics), Governance & Monitoring (three topics), Procedural (three topics), and Epistemological (three topics).

The Principle theme is concerned with the ethical constructs of artificial intelligence, whereas the Personal Data Protection theme is concerned with the protection of personal data in digital environments. The Governance & Monitoring theme is concerned with the methods of AI surveillance and governance. The Governmental theme refers to the use of AI in government, particularly in decision-making. The Procedural theme is concerned with AI design and development procedures such as data processing, design, and ethical learning. The epistemic attributes of artificial intelligence systems, such as data models, data training, or algorithms, are associated with the Epistemological theme.

### Qualitative Analysis of Topics

In this section, we conducted qualitative analysis of policies to go beyond simply identifying policy topics. This analysis entails a more in-depth engagement with the policy documents in order to identify not only subjects the policies are addressing (the topics), but also how they are addressed (the underlying themes and their interpretation). This integration is critical for our analysis because it helps us understand not only the presence of ethical topics in AI policies, but also their importance and the intent behind their inclusion. We aim to provide a comprehensive picture that includes both objective delineation of topics and qualitative analysis of their treatment within policy documents by examining both the frequency of terms and their contextual use. This approach aims to provide readers with a more nuanced understanding of the AI policy landscape.

#### Ethical Principles of Artificial Intelligence

The first thematic component is concerned with AI's ethical principles. This includes aspects such as accountability, responsibility, safety, transparency, trust, and accountability, as well as democratic values and shared common goods.

##### Responsible AI Systems

The first thematic component, “Principles,” delves into the ethical concepts of AI. Responsible AI systems have emerged as a central topic in government discussions about ethical AI. The emphasis is not only on the systems themselves, but also on scrutinizing automated systems for potential liability, accountability, and responsibility. Identifying the individuals and organizations responsible for the creation and deployment of AI algorithms is a critical component. Another major concern is the creation of AI systems that adhere to pre-existing social and cultural norms. This includes ensuring the safety of AI systems while protecting individual privacy and transparency. This theme also includes a discussion of the spread of fake news and disinformation by untrustworthy automated decision-making systems. Furthermore, the theme delves into algorithmic governance, which refers to the use of AI-based technologies to assist governments in making decisions. Concerns have been raised about the government's inadequate handling of the issue, increasing fears of a loss of human competence within agencies, a widening of the public–private technological divide, less transparency in public decision-making, and increased public apprehension about arbitrary government action and authority. The theme also addresses the importance of innovative and agile governance structures as well as risk frameworks. These are presented as useful in guiding AI governance teams and should be continuously optimized in collaboration with a wide range of stakeholders. The theme also addresses governance innovation, which entails redesigning laws and architecture to meet the social changes brought about by digital technologies. The theme also delves into the idea of participatory governance, which entails direct citizen participation in the shared and collaborative administration of open-access digital goods. The theme does not advocate for restricting AI use, but it does stress the importance of proper data governance of AI-enabled capabilities in order to avoid the risks posed by incorrect and biased datasets. It suggests designing algorithms with data governance in mind. The theme also emphasizes the creation of governance principles for new and complex AI systems such as self-driving cars and precision medicine, demonstrating the breadth and complexity of the issues at hand.

##### Safety of Autonomous Vehicles

The theme of "Principles" also includes the topic of self-driving and connected cars, as well as the safety concerns, they raise. Autonomous vehicles, according to ongoing discussions in government documents, require safety oversight and regulation. The discussion emphasizes the importance of an independent testing facility and appropriate stakeholder organizations, such as consumer watchdogs, to oversee the development of self-driving vehicles. The testing of self-driving cars is highlighted in this context. This includes testing in both laboratory settings and specialized test tracks, as well as regulated real-world conditions. There is also discussion regarding increasing vehicle transparency by having a better understanding of the risks and opportunities. The installation of data recording devices with automated functionality in test cars is emphasized heavily. This allows data from sensors and control systems to be recorded alongside the vehicle's motion. The topic implies that automated and linked systems licensed and installed on public roads must be monitored by the government to ensure their safety.

##### Transparency, Trust, and Accountability (TTA)

The theme of "Principles" extends further to the development of an open, trusting, and accountable culture in the establishment of new regulatory frameworks. According to the topic, an AI-ready society ensures justice, transparency, and accountability in decision-making, as well as fosters trust in the technology. Its goal is to ensure that people who use AI do not face unfair treatment because of their personal backgrounds or a lack of respect for human dignity. The creation of a system for assessing companies' compliance with their duty of care and improving representation at all levels of decision-making is a key focus. This topic proposes that companies be required to submit Transparency, Trust, and Accountability (TTA) reports on an annual basis. These reports would show how businesses are dealing with harmful content and preparing for it. The topic also delves into the intricate relationships that exist between bias, fairness, and transparency and AI-enabled predictive systems. Because AI-enabled prediction systems can improve repeatability and reduce human error and bias, they are fraught with moral problems. Indirect discrimination and transparency are examples of these. Examining the transparency and accountability of these systems will help to understand how predictions are generated and, thus, how bias can be eliminated. Furthermore, the topic suggests solutions that promote collaboration, teamwork, and shared responsibility, such as accountability and transparency. These approaches are presented as critical to fostering a positive culture around AI system implementation.

##### Human Autonomy and Social Justice

The "Principles" theme delves deeper into the interconnectedness of autonomy, privacy, and the concept of personal accountability. When confronted with issues of technological determinism and tool dependence, AI development should promote human autonomy. Rather than humanizing AI, the topic emphasizes the importance of respecting human dignity and autonomy while managing AI responsibly. Autonomy can be defined in a variety of ways, the most common of which are a person's moral, political, and functional autonomy. These include the autonomy of a disabled AI-assisted person, the autonomy of an AI-populated environment, and the autonomy of AI in a human-populated environment. AI systems should not infringe on human autonomy (moral, political, and functional); rather, they should enhance it. According to government documents, AI systems should not become fully autonomous and should instead be subject to human supervision (moral, political, and functional). As a result, autonomy should not take precedence over other considerations such as justice or the general well-being of society. According to government documents, AI service providers and corporate consumers should respect human dignity and individual autonomy while employing AI. The approach emphasizes the importance of personal accountability in the use and development of AI systems.

##### Common Goods and Democratic Values

The "Principles" theme also addresses the common goal of promoting democratic AI use for collective benefit in democratic societies. It emphasizes adhering to democratic principles while mitigating the risks of rights and interests’ violations during AI use. The topic emphasizes how AI's ethical norms and ideals are still based on generic and abstract concepts. Government documents propose initiatives such as algorithmic governance, digital literacy, digital inclusion of diversity, and ecological sustainability to harness AI for the common good. As a result, it advocates for the use of personal data in democratic contexts that take accountability and the common good into account. This topic also addresses the dissemination of disinformation and falsehood by AI, as well as the politicizing of social media in promoting harmful misinformation that undermines democratic ideals and principles. This topic examines whether the goals and applications of AI are ethical, socially responsible, and consistent with democratic norms. It emphasizes the importance of being cautious and proactive in ensuring that AI technologies align with societal values and principles.

#### Personal Data Protection

##### Informed Consent and General Data Protection Regulation

The theme of "Personal Data Protection" focuses on Informed Consent and the General Data Protection Regulation (GDPR). Algorithmic decision-making is a critical issue within this theme, centered on individuals' explicit, independent, free, and informed consent to the use of algorithms. One major source of concern has been the inadequacy of the consent protocols used for data collection, access, and sharing. The topic discusses difficult scenarios in which an individual is unable to express consent or their consent is legally invalid due to physical or mental incapacities. Consent must be freely given, specifically in an informed and transparent manner, and must be expressed by individuals taking clear affirmative action. Individuals should ideally be able to reclaim control of their personal data; however, this can be difficult if they are required to make decisions that are outside of their knowledge and experience. Furthermore, the data controller is required to put in place adequate safeguards to protect the rights, freedom, and legitimate interests of those whose personal information they hold. The data controller may not continue to process the data unless the data subject provides explicit consent. The GDPR is also a major focus of government documents. This regulation is crucial in shaping data protection standards and practices, ensuring that personal data is handled in a way that respects individual rights and liberties. The GDPR's data minimization, purpose limitation, and accountability principles are critical in this context.

##### Children Online Safety

The theme of "Personal Data Protection" also includes "Children's Online Safety." This topic focuses on children's safety and protection in online environments or AI systems such as facial recognition or digital identity systems. The topic supports less disruptive forms of identification in schools and safer design standards for children in the online world. Advocacy also extends to preventing the use of children's personal information for purposes such as behavioral advertising or recommendation systems. Exposure to harmful content on platforms such as Facebook and other social media platforms, games, and various forms of online media can have a negative impact on children's and adolescents' mental health and well-being. Despite being the most vulnerable to online harm, children are often less likely to receive guidance and education on how to protect themselves. In order to empower users and maintain a free, open, and secure internet, this topic promotes initiatives such as online safety education and awareness-raising, as well as the multi-stakeholder model of internet governance. The subject also investigates the potential of technologies such as AI and machine learning, as well as hashing and fingerprinting, to automatically detect harmful content in online spaces. There is also discussion about Safety by Design frameworks, which suggest a way for organizations to integrate online safety into their digital offerings from the very beginning. These frameworks can assist in ensuring that products and services are designed with user safety in mind, with a focus on vulnerable user groups such as children.

#### Governmental

##### Machine Assisted Human Decisions

The "Governmental" theme starts with the topic "Machine Assisted Human Decisions." This topic delves into the complexities and difficulties that arise when humans rely on AI systems to make decisions. Automated decision-making can be useful in situations where numerous decisions must be taken depending on specific criteria. Such automated decision-making systems could be used to help individuals reach their own decisions. Accountability is a key component of this discussion, as concerns arise about the complexities of liability when an automated administrative decision goes wrong. As stated in this topic, humans must accept responsibility for automated systems' decisions. Exceptions to this rule may include situations in which human judgment is unnecessary, such as in certain administrative decisions. However, when making legal decisions, government officials should avoid using automated decision-making systems. This reflects the principle that significant decisions affecting individuals' rights or freedoms should remain under human control. This topic also touches on algorithm-based recommender systems, which provide consumers with recommendations such as restaurants. Given the 'black box' nature of AI algorithms, it asserts that these algorithms must still be subject to human oversight and should operate transparently. This fosters trust and understanding of these systems, ensuring their responsible and ethical use.

##### Automated Decision-Making (ADM)

"Governmental" theme's second topic is "Automated Decision-Making (ADM)." This topic's central argument involves the connections between bias, discrimination, and the use of Automated Decision-Making and algorithmic processes. According to the topic, robotic systems have the potential to reduce bias and protect privacy. However, there is a danger that automated decisions will increase bias and discrimination. As a result, strategies for improving input values and sampling methods are proposed to reduce the occurrence of bias. A significant issue raised in this topic is the possibility that providing a detailed explanation for each step of an algorithm may jeopardize the system's performance. The term "automation bias" refers to the tendency to overlook alternatives to a computer-generated result that is widely accepted as correct. The lack of consistent and accurate outputs from automated decision systems in the case of facial recognition could contribute to societal bias. The debate here revolves around the creation of new legal frameworks and anti-discrimination laws to oversee algorithmic decision-making. The goal is to ensure fairness, accountability, and transparency in these systems while mitigating any negative consequences.

##### Algorithmic Law Enforcement

"Algorithmic Law Enforcement" is a subtopic of the "Governmental" theme. This topic focuses on governing and enforcement functions such as police, civil enforcement, criminal enforcement, and regulatory analysis. This topic also delves into issues of accountability and conflict caused by AI-related law enforcement, as well as threats to the transparency and democratic accountability of law enforcement organizations. It contends that public acceptance and trust are critical to the success of AI in law enforcement. Furthermore, it highlights the importance of AI regulations addressing the ethics of using real-time remote biometric identification or emotion detection in public settings for law enforcement purposes. Such practices may violate the right to privacy, requiring changes to administrative law principles. This section also discusses data protection laws as well as law enforcement's response to crime on the dark web and the internet. According to the topic, effective algorithmic enforcement tools necessitate training data that accurately reflects the truth about wrongdoing. The government documents emphasize that instead of relying on third parties and the commercial sector, enforcement agencies should focus on developing their internal capacity and regularly updating their algorithms and systems. This approach is intended to ensure that external entities cannot compromise the security of AI-based enforcement tools. The topic also addresses law enforcement personnel's mistrust of algorithmic enforcement and the need for explainable output. Furthermore, it addresses political concerns about a digitalized enforcement agency, emphasizing the importance of striking a balance between technology-enabled efficiency and public trust and accountability.

#### Governance & Monitoring

##### Responsible Private Sector and Targeted Advertisement

The first theme under "Governance & Monitoring" is "Responsible Private Sector and Targeted Advertisement." This topic emphasizes the commercial sector's obligation to prevent the dissemination of their customers' personal information for purposes such as targeted advertising or the dissemination of harmful, illegal, or terrorist content. It also addresses concerns about targeted advertising based on internet usage. According to the topic, websites should inform users about the data they collect and how it is used. These platforms can also be used to target specific customers with commercial product or political campaign advertisements. The emphasis is on how to build trust between consumers and businesses while balancing intrusive and beneficial targeted advertising. The topic also addresses concerns about the proliferation of terrorist and extremist content on the internet. It emphasizes the claim that corporate efforts to improve security have been slow and inconsistent. Businesses should develop solutions to help customers avoid information or behavior that encourages suicide or self-harm. According to the topic, governments ought to establish new legal obligations in order to encourage private companies to take on more responsibility and agility in protecting their customers. They should also be able to compensate customers for losses caused by the content or activities of their services. This strategy aims to make the internet a safer and more responsible place for all users.

##### High Risk Assessment and Impact Analysis

"High Risk Assessment and Impact Analysis" is a topic under the "Governance & Monitoring" theme. The emphasis here is on high-risk AI services. Given the potential risks that AI may pose to vulnerable groups, this topic emphasizes the importance of thorough risk assessments and monitoring. It classifies AI systems as high-risk when they handle biometric data, such as biometric identification and classification of natural individuals. A high-risk AI system is one that has the potential to endanger people's health and safety or impair their fundamental rights. The primary question, as illustrated by this topic, is how to measure and evaluate a negative impact. In addition to regular, systematic assessments and updates, high-risk AI systems necessitate the following: (1) Identification and assessment of known and anticipated hazards associated with each high-risk AI system. (2) Assessments and evaluations of the risks that may arise when using the high-risk AI system. (3) Identification of additional potential risks based on data from post-market monitoring systems. (4) The implementation of appropriate risk management measures. In the context of this topic, data controllers must conduct a Data Protection Impact Assessment (DPIA) to determine how their proposed processing activities will impact personal data protection. Furthermore, the topic is concerned with assessing the impact of high-risk AI systems on fundamental rights. The goal is to ensure that the implementation of these systems does not jeopardize people's safety, privacy, or other rights.

##### Civil Society (Multi-stakeholder) Participation

"Civil Society (Multi-Stakeholder) Participation" is a subtopic of the "Governance & Monitoring" theme. This topic emphasizes the importance of involving citizens, civil society, and other stakeholders in the design and development of AI systems. The topic addresses issues such as how much AI risk is acceptable, as well as how to encourage ethical innovation and the development of safety technologies such as safety-by-design. The discussions, as the theme emphasizes, will promote responsible innovation and aid in the establishment of strong, trustworthy governance institutions. Stakeholders should be held accountable for how they use AI. The topic emphasizes the importance of independent entities such as advisory bodies in connecting civil society to other stakeholders. They can assist in the development of appropriate governance regimes or host conferences or workshops for civil society. This involvement will encourage the inclusion of diverse ideas in the creation and design of AI systems. However, the topic acknowledges skepticism and disagreements about the complexity of these algorithms, which may be beyond the general public's comprehension. This difficulty is prominently mentioned in government documents, emphasizing the importance of transparent and understandable AI systems.

#### Procedural

##### Privacy by Design

"Privacy by Design" is a topic under the "Procedural" theme. It focuses on the concept of "Ethical by Design," which includes subcategories such as "Privacy," "Security," and "Safety by Design." This topic promotes user rights and interests by encouraging AI system developers to identify and mitigate risks during the design process. The term "Human-Centered Design" is highlighted, promoting diversity as a design approach that limits the interests of individual stakeholders while reducing bias in input data. The topic contends that all parties involved in the development of AI systems should conduct risk and impact assessments. This approach ensures that the AI system's potential effects and risks are thoroughly analyzed and mitigated to the greatest extent possible before deployment. Furthermore, this topic promotes making an AI system's design process public for audit and external criticism. This transparency can help identify potential issues that the development team may have overlooked, and it contributes to the AI system's overall trustworthiness and credibility. The government documents emphasize the importance of transparency and public scrutiny in ensuring that AI systems respect user privacy and operate ethically.

##### Controllers of Personal Data Processing

The topic "Controllers of Personal Data Processing" is part of the "Procedural" theme. This topic focuses on the role of data controllers in the processing of personal data, particularly in the context of automated decision-making. Data controllers are responsible for defining the purposes for which personal data will be processed and ensuring that these purposes are consistent with the original reasons the data was collected. This topic's key elements include preliminary impact assessments on data protection and the necessary disclosures to data subjects about automated decision-making. Because of the power imbalance between data controllers and data subjects, current data protection standards may not be suitable for remote biometric identification systems. The topic implies that discussions about fairness should propose solutions to this problem. If no legitimate reasons for processing can be identified, the design phase of processing cannot proceed. The General Data Protection Regulation (GDPR) imposes these criteria on controllers and emphasizes the importance of validity throughout the design phase of processing. The overarching goal is to ensure that personal data is handled in a way that respects individual privacy while adhering to legal and ethical standards.

##### Ethical AI Talent Workforce

The topic "Ethical AI Talent Workforce" belongs to the "Procedural" category. To minimize ethical implications, this topic emphasizes the importance of educating data scientists, machine learning model developers, and those working with algorithms. The topic contends that if AI is developed without the application of critical thinking and evaluations by the AI workforce, problems may arise. For example, in automated driving, test drivers and operators must have skills that go beyond those required of typical drivers. This implies that in order to provide a safe and effective test environment, engineers and developers must have a thorough understanding of the capabilities and limitations of the technologies under test, as well as the associated risks. The topic focuses on educating people to be ethical, democratic, and pragmatic users of new technology. It also discusses the transdisciplinary nature of AI, its consequences, and the importance of lifelong learning in educational systems. Furthermore, the topic addresses the gender gap in digital skills and the low representation of women in the field of AI. While AI and automation are expected to transform skills across professions, those with less education are likely to be impacted the most by the transition. As a result, the topic implies that both vocational and higher education require reform. This topic also emphasizes the importance of inclusive and accessible digital literacy education in order to reduce barriers to access, improve knowledge sharing, and encourage underrepresented groups to actively participate in digital inclusion. The ultimate goal is to develop a diverse and ethical AI workforce that values democratic values and the rights of all individuals.

#### Epistemological

##### Data Models & Training Data

"Data Models & Training Data" is a subtopic of the "Epistemological" theme. This topic emphasizes the significance of diverse data sources and training algorithms, while also raising concerns about non-resilient and non-inclusive training data, which may contribute to bias and discrimination in AI outcomes. The topic contends that using biased training data in an AI model would only contribute to maintaining existing social injustices. It emphasizes the importance of data subject feature selection, as different features in a training data set can produce different results. While the topic discusses feature selection approaches commonly used by data scientists that may result in data reduction, it also points out that these approaches are frequently marginalized. The topic emphasizes that training data are susceptible to inherent bias, may not be representative of individuals, and may not contain a set of norms and values. Poor data quality may also result in bias. The precision of outputs is directly related to the quantity and quality of training data, such as facial images. This topic also addresses issues such as the lack of Asian faces in training datasets, which causes problems with eye recognition for people of Asian descent. There is also discussion of the lack of training data and technology in poor and developing countries, as well as the need for government transparency in training data. Furthermore, this topic warns about the potential problems associated with a large quantity of training data. Overadjustment and overfitting may result in an overfit model, making it more vulnerable to privacy attacks. This topic emphasizes the importance of careful balance and management in the use of training data in order to create accurate, fair, and secure AI models.

##### Explainable AI

"Explainable AI" is a subtopic of the "Epistemological" theme. It focuses on AI systems' ability to generate high-quality explanations. Explainability can be defined in several ways, including whether the system produces an explanation that humans can understand, whether the explanation accurately depicts the system's processes, and whether the system communicates its knowledge boundaries. In the case of non-sensitive public sector applications, AI operators may consider making the source code and an explanation of how the system works public or available upon request. In practice, however, auditing a machine learning system can be difficult. This topic raises concerns about how simple it is to explain the complex inner workings of AI systems. Many algorithmic decision tools are not structurally explainable, raising concerns about the amount of transparency and explainability required. Explainable AI's goal is to make AI decisions more understandable and trustworthy. The challenge, however, is to strike a balance between the complexity and accuracy of AI models and their interpretability and explainability. This topic emphasizes the significance of continuing the debate and research in this area in order to achieve this balance.

##### Bias, Discrimination, and Fairness of Algorithms

The topic "Bias, Discrimination, and Fairness of Algorithms" is part of the "Epistemological" theme. It focuses on bias, discrimination, and fairness in algorithms, machine learning models, and datasets. Bias can be introduced by using measurements, features, or data that do not accurately represent fairness, or by not employing metrics that adequately assess fairness during the monitoring stage. Datasets, as discussed in government documents, may intentionally or unintentionally exclude certain groups of people, resulting in a lack of diversity. Throughout the AI lifecycle, various stakeholders, such as algorithm developers or government agencies, are held accountable for mitigating bias in AI systems. Although there is no universal agreement on the criteria for measuring and auditing bias in AI systems, bias auditing technologies are being developed and patented to analyze algorithmic discrimination. The discussion also includes both direct and indirect discrimination. When a model includes data variables that are closely related to the variables used for discrimination, indirect discrimination can occur. For example, even if an algorithm does not include race as a factor in its model, it may discriminate against a neighborhood made up entirely of people of one race, resulting in racial outcomes. As a result, the significance of feature engineering in preventing indirect discrimination is highlighted. When applying bias mitigation techniques to AI models, there may be trade-offs between fairness and model accuracy or robustness. Another issue raised by this topic is the possibility that extending data and identifying patterns will result in inaccurate predictions of future behavior and events. As a result, this topic emphasizes the importance of careful thought and ongoing discussions about bias, discrimination, and fairness in the development and application of AI systems. It advocates for more research, transparency, and regulation to ensure that AI systems are fair and unbiased.

### Distribution Analysis of Topics Across Countries

In this study, the distribution analysis feature of Word Stat was used to examine how discussions about key ethical AI issues vary geographically. The analysis illuminates how local contexts influence the focus on different ethical AI topics, as well as the regulatory environment. The emphasis on "Responsible AI" in policies from nations such as the USA, India, UK, Japan, and Canada indicates a concerted effort in these countries towards ethical AI development. Conversely, the lower incidence of these topics in places like Dubai and China may point to reduced engagement or interest in these facets of AI ethics. When it comes to thematic engagement, leading AI nations including the USA, Canada, India, UK, and China, are involved with a broader range of topics, probably because of their expansive AI sectors and research networks. Issues like privacy, data protection, and informed consent are widely addressed, likely in response to international regulations like the GDPR. The focus on bias, fairness, and impact analysis underscores the global importance of these concerns. Looking at the disparities, the USA shows the highest level of interaction with nearly all topics, aligning with its status as a pioneer in AI. European countries, particularly the UK, Germany, France, and the Netherlands, are also prominent in addressing these issues, reflecting their regulatory regimes and ethical priorities. Nordic nations like Finland and Norway show a strong involvement in certain areas (e.g., workforce, public participation), indicative of their societal frameworks. In Asia, there's a varied picture; China participates broadly but is less active on delicate topics like autonomy and enforcement, while Japan's engagement is inconsistent across issues. Among developed countries, Canada stands out for its comprehensive engagement, whereas Australia and New Zealand are less active on some topics. Smaller countries with less AI activity overall may still show significant involvement in specific areas, as seen with countries like Belgium and Ireland.

## Analysis

To determine the most prevalent and important themes and topics in government ethical AI publications, we conducted a mixed method analysis of national policy documents, combining quantitative topic modeling and qualitative content analysis. Based on the topic modeling analysis, the software extracted 19 topics, which the authors then classified into six broader themes using thematic synthesis. These were the AI Principles, personal data protection, government, governance and monitoring, procedural and epistemological issues.

The ethical AI principles theme underscores the importance of establishing governance frameworks that ensure transparency, accountability, safety, and the safeguarding of human values. Such measures are crucial for ensuring that AI develops responsibly and achieves its full potential. Innovative regulatory frameworks and supervision mechanisms that promote collaboration, independent testing, risk assessment, and reporting are imperative in various domains, including autonomous vehicles, predictive systems in government, and social media applications. Regulation of algorithms and data practices is necessary to safeguard privacy, advance justice, and facilitate democratic decision-making. In general, for ever more intricate AI systems, the development and implementation of AI technologies should uphold sustainability-focused governance concepts, provide algorithmic training, and promote ethical and socially responsible conduct. Such endeavors ought to protect human autonomy and dignity, advance the common good, and support democratic norms and principles, as opposed to undermining them.

Topics comprising the second overarching theme of "personal data protection" underscore pivotal concerns such as obtaining informed consent, protecting data, and ensuring online safety in the context of algorithmic and personal data usage. It is imperative to implement appropriate protocols, such as ensuring compliance with the GDPR principles for data controllers and verifying that explicit consent is voluntarily provided in accordance with an individual's capacity. Especially with regard to vulnerable populations such as children, this is critical. Safe design principles are required due to their potential exposure to AI systems and the online environment. These principles prohibit the utilization of their data for detrimental objectives, such as behavioral advertising. Preserving their mental health and online safety is of the utmost importance. Technological advancements that implement a "Safety by Design" approach across all online services may facilitate the recognition of inappropriate content. In order to enhance public trust in the responsible development of algorithms that utilize personal information, protecting sensitive demographics and consent protocols are integral components.

The third theme, governmental, delves into substantial concerns surrounding the implementation of automation and algorithms in the process of human decision-making, specifically in circumstances that have legal or social consequences. When criteria are well-defined, machine assistance can be beneficial and aid in reducing biases; however, there are concerns that accountability, bias, and inconsistent outputs could perpetuate societal discrimination if not implemented with care. New frameworks are necessary to address issues such as "automation bias" and the regulation of automated decision-making systems. This holds especially true in delicate domains such as law enforcement, where accountability, transparency, public acceptability, and data protection are critical in light of the democratic values threatened by adversarial, opaque algorithmic policing. It is recommended that appropriate legal and administrative changes, reliable training data, and internal agency capacity building be executed to ensure that AI augments human judgment rather than diminishes it when authoritative individuals make critical decisions.

Governance and monitoring encompass substantial topics such as risk assessment, ethical data usage, and public participation in AI systems. Ensuring appropriate precautions is crucial in various domains, including the private sector's responsibility to prevent detrimental targeting and improper handling of customer data, the comprehensive risk assessment of critical applications, and the facilitation of productive engagement with civil society. It is critical to conduct exhaustive risk assessments pertaining to privacy, security, and rights, both known and potential. Design adjustments and legislation can aid in protecting users and repairing damage. Advisory bodies that operate independently may foster dependable governance and facilitate comprehension. Responsible innovation and the proper balance of public, private, and civic interests will generally require the following: Safety by Design, corporate accountability, regular risk transparency, and multi-stakeholder participation in decisions regarding acceptable risks.

The procedural topics cover fundamental concepts for developing AI in an ethical manner for the duration of its lifecycle. A holistic strategy is required that addresses all angles, such as educating the public to be ethical and knowledgeable about the risks posed by artificial intelligence, clarifying the duties of data controllers in handling personal information, and incorporating principles of "ethical by design" into system design from its inception. Key elements include the implementation of risk assessments, the facilitation of public scrutiny, the adherence to data protection regulations, and the mitigation of power imbalances and prejudices. It is necessary to educate the workforce to evaluate acceptable applications of technology critically and to understand its potential and limitations. Finally, by reevaluating education and advocating for digital literacy, a diverse workforce can be cultivated that can mitigate AI's impact through multidisciplinary, risk-aware design processes and continuous evaluations that value users' perspectives and consider societal consequences.

Epistemological topics underscore the importance of exercising caution when selecting the training data for AI models, so as to construct systems that are both equitable and inclusive. The data utilized for training purposes ought to be diverse, unbiased, and representative of the population. However, inherent biases are difficult to quantify and eradicate. Diverse regions and countries focus on distinct societal issues according to their respective requirements and priorities. Explainability, bias auditing, accountability throughout the AI lifecycle, high-quality data sources, and bridging global data access disparities are all elements that responsible AI development must consider. In order to mitigate biases in decision-making processes, models, and data through international multi-stakeholder consultation, cross-border collaboration, knowledge exchange from diverse governance best practices, and consideration of distributional effects will be crucial.

## Discussion

The Principles theme comprises six interconnected topics: democratic principles, responsible AI systems, autonomous vehicle safety, transparency, trust, and accountability; human autonomy; social justice and common goods; and transparency. These topics collectively constitute a comprehensive framework for AI ethics. Fundamentally, the responsible AI system serves as a cornerstone principle that mandates the development and implementation of all AI technologies in accordance with standards of integrity, confidentiality, and fairness. An example of how these foundational principles are refined and placed into context in practice is the safety of autonomous vehicles, which is a particular implementation of AI (Hengstler et al., 2016). One way in which AI practitioners cultivate a culture of accountability is through the implementation of transparent decision-making processes (Textor et al., 2022). The preservation of human agency, specifically in AI-assisted decision-making processes, is a connection between these ideas and human autonomy. Democratic values and social justice expand the domain of AI ethics beyond the individual to encompass society at large. This broadens the potential of AI as a tool for promoting social welfare while also cautioning against its potential misuse, which may compromise democratic procedures and exacerbate social disparities (Cowls et al., 2021). Therefore, these subjects establish an ethical framework for AI that is both pragmatic and aspirational, striking a balance between the practical constraints of AI implementation and a more extensive societal obligation to justice, democracy, and the collective wellbeing.

The interconnection between informed consent, the GDPR, and the online safety of children is crucial, as it illuminates the discourse surrounding data privacy and digital ethics (Saheb, 2020). A fundamental prerequisite of the GDPR is informed consent, which stipulates that individuals handling data must not only possess knowledge of the collection, processing, and storage operations but also comprehend their inherent characteristics and consequences (O'Connor et al., 2017). When considering the online safety of children, the concept of informed consent assumes particular significance. Due to their limited capacity to completely comprehend and provide informed consent regarding the complexities of data processing, children, who are considered a vulnerable group, necessitate supplementary safeguards. The protection of the child provision of the GDPR stipulates that in the case of online services accessed by minors under the age of 16, parental consent is mandatory (Slabu, 2017). These concerns underscore the critical significance of empowerment, transparency, and rights protection within the digital ecosystem, with a particular emphasis on the most marginalized individuals.

The concepts of algorithmic law enforcement, human-assisted decision-making, and automated decision-making are interrelated and signify the progression of AI and machine learning technologies within the realm of decision-making. Human decision-making is enhanced using data-driven insights made possible by AI technologies in a cooperative model known as machine-assisted human decisions (Prabhudesai et al., 2023). The notion is associated with automated decision-making, wherein computers make judgments independently with minimal or no involvement from humans. These principles find their physical expression in the domain of algorithmic law enforcement, which pertains to public order and law. Both concepts are fundamental to this field. Utilization of both machine-assisted judgments (e.g., predictive policing, where AI notifies human officers of potential crime areas) and automated decision-making (e.g., the automated imposition of traffic penalties on the basis of AI surveillance systems) is encompassed in algorithmic law enforcement. Although these technologies may streamline operations and increase productivity, they also raise significant apprehensions regarding accuracy, bias, openness, and responsibility (Pastaltzidis et al., 2022). These themes thus underscore the critical necessity for ethical and legal protections and reflect the revolutionary impact of AI on our decision-making systems.

At the juncture of technology, society, and ethics, three interrelated challenges stand out: responsible private sector conduct, specifically in regard to targeted advertising; civil society engagement; and high-risk assessment and effect analysis. Civil society participation is of the utmost importance in the digital age for fostering a democratic discourse on technology usage and ensuring that policies pertaining to technology consider a wide range of societal viewpoints (Reia, 2022). Particularly in regard to activities such as targeted advertising, this participation is vital for holding the private sector accountable for responsible conduct. Although data-driven advertising strategies offer certain benefits to businesses, they also give rise to significant ethical considerations, including infringements upon privacy and the possibility of manipulation (Saura et al., 2021). Critical methods for evaluating these practices are high-risk assessment and impact analysis, which examine the potential dangers and broader societal repercussions presented by such technology. They play a crucial role in the regulatory framework by aiding in the detection and reduction of potential risks. By ensuring that digital technologies and their applications adhere to democratic norms, ethical standards, and social expectations, these concerns collectively underscore the significance of a multi-stakeholder, participatory approach to their development.

The idea of "privacy by design," the roles of controllers in handling personal data, and the creation of an ethical AI talent pool are all related topics that help reach the main goal of ensuring ethical and privacy-conscious actions in AI and data science. The responsibility of identifying the objectives and methods of processing personal data, as well as assuring adherence to data protection legislation, falls on controllers of personal data processing (Lindqvist, 2018). This requirement is intrinsically tied to the concept of privacy by design, which advocates for privacy to be included into system design from the start rather than as an afterthought (Rubinstein, 2011). Adopting a proactive stance towards privacy guarantees that the architectural foundation of AI systems incorporates data protection principles, thereby fostering confidence and adherence to regulatory requirements. A workforce of ethical AI professionals is necessary for the development of both of these facets. Building a culture of ethics and privacy awareness among AI professionals is important to make sure they can make smart decisions about data processing, system design, and general operations (Ryan et al, 2022). These viewpoints underscore the necessity for a comprehensive strategy in tackling privacy and ethics within the exponentially expanding AI ecosystem.

The domain of ethical AI encompasses the interconnected concepts of data models and training data, explainable AI, as well as bias, discrimination, and impartiality in algorithms. Data models—mathematical structures employed to depict the interconnections among variables in data—and the training data utilized to construct these models—form the bedrock of AI systems. According to Salzz (2019), the outcomes generated by AI systems can be significantly influenced by the diversity, quality, and representativeness of the data. Particularly in high-stakes domains, explainable AI (XAI) is crucial for assisting stakeholders in comprehending how AI systems arrive at decisions. The ability to detect and rectify bias, discrimination, and injustice in algorithms is contingent upon this transparency. These concerns often emerge due to biases present in the training data or mathematical assumptions made in the data models. Improper handling of these issues may lead to discriminatory or unjust outcomes. Collectively, these issues underscore the significance of fairness, interpretability, and transparency in the development and deployment of AI systems, thereby emphasizing the need for rigorous standards and ethical deliberations in AI research and implementation.

The findings of this analysis shed light on the variation in regional priorities regarding significant ethical issues in AI. This underscores the intricate relationship between local conditions, regulatory environments, and the discourse surrounding AI ethics (ÓhÉigeartaigh et al., 2020). The considerable prominence attributed to "Responsible AI" in policy frameworks of nations including the United States, India, the United Kingdom, Japan, and Canada signifies a robust commitment to the development of ethical and responsible AI. However, the comparatively lower counts observed in China and Dubai suggest a possible divergence in interest or priority regarding these particular facets of AI ethics. It is probable that the global ramifications of regulations such as the GDPR have sparked considerable interest in subjects like informed consent, privacy, and data protection in numerous countries. Similarly, the universal acknowledgement of these issues is demonstrated by the extensive attention paid to biases, fairness, and impact analysis. Nevertheless, regional disparities are evident, as the United States assumes a prominent position in the majority of matters, a reflection of its influence as a global leader in artificial intelligence, while European countries consistently make substantial contributions due to their robust ethical and regulatory systems. The different levels of engagement among Asian and developing countries, as well as smaller states, emphasize the complicated ways in which regional contexts and AI capabilities influence ethical AI discussions. The results underscore the importance of comprehending regional discrepancies in the ethical treatment of AI. It also contends that efficient worldwide regulation of AI would necessitate the recognition and handling of these inequities.

## Limitations of the Study and Possible Future Studies

While this study provides useful insights, it is not without limitations. The analysis was limited to English-language policies, which may have excluded significant policy developments in non-English speaking regions. As a result, future research could aim to bridge this linguistic gap by investigating ethical AI policies articulated in other languages. Furthermore, our work primarily focused on a policy review of ethical AI. Future studies could conduct a more in-depth examination of the broader technology policy landscape. Such efforts would broaden our understanding of the multifaceted implications of AI and other emerging technologies, enriching the discourse on technology policy analysis.

## Conclusion

To summarize, developing AI responsibly and for the benefit of humanity will necessitate a comprehensive approach that takes into account its entire socio-technical context and implications. Responsible policies must address key issues such as governance, oversight, data protection, transparency, bias mitigation, human autonomy, privacy, education and workforce preparedness, multi-stakeholder collaboration, and distributional impacts. While progress is being made in some areas and domains, the analysis reveals gaps that must be filled. A comprehensive framework that considers the entire data, system, and policy lifecycles is required. International cooperation on best practices can aid in the development of AI that is inclusive, safe, and trustworthy while adhering to shared democratic principles and human rights. Ongoing oversight and assessment will also be necessary to ensure that governance structures continue to evolve to meet new challenges in a complex, fast-paced field.
