# Correction: Mapping Ethical Artificial Intelligence Policy Landscape: A Mixed Method Analysis

**DOI:** 10.1007/s11948-024-00484-2

**Published:** 2024-05-22

**Authors:** Tahereh Saheb, Tayebeh Saheb

**Affiliations:** 1 School of Business, Menlo College, Atherton, CA USA; 2 Faculty of Law, Tarbiat Modares University, Tehran, Iran

**Correction: Science and Engineering Ethics (2024) 30:9**

10.1007/s11948-024-00472-6

In this article Prof. Tayebeh Saheb at affiliation Faculty of Law, Tarbiat Modares University, Tehran, Iran was missing from the author list.

The original article has been corrected.

